# Supplementary material for: Undiagnosed Diabetes and Prediabetes in Patients with Chronic Coronary Syndromes—An Alarming Public Health Issue
Source: J Clin Med. 2021 May 5;10(9):1981. doi: 10.3390/jcm10091981 (PMC8124594; doi:10.3390/jcm10091981)
Supplement: Supplementary file 1 [file jcm-10-01981-s001.zip › jcm-1133020-supplementary.pdf]

Supplementary data

In our study group the diagnosis of DM, IFG or IGT have been made according to WHO criteria (Table S1).

Table S1. Values for diagnosis of DM and other categories of hyperglycaemia.

	**Fasting mM (mg/dL)**	**2h Post Glucose Load mM (mg/dL)**
DM	DM was not diagnosed on the basis of fasting glucose due to availability of a single measurement	≥11.1 (≥200)
IGT	No previous DM diagnosis	≥7.8 (140) and < 11.1 (200)
IFG	5.6 (100)–7.0 (126)	< 7.8 (140)

A summary of distribution of dysglycaemia in the examined group is included in Table S2.

Table S2. Distribution of glucose metabolism disturbances in patients with OGTT performed (n = 546).

		**IGT**	**Newly Diagnosed DM**
**Fasting Glucose (mg/dL)**	<100	43 (7.9%)	5 (0.9%)
	100–126	92 (16.8%)	12 (2.2%)
	≥126	26 (4.8%)	11 (2.0%)

Table S3. Univariate Multinomial Logistic Regression: reference category Normal Glucose Tolerance (NGT).

		Prediabetes (IFG or IGT)				DM after hospitalization			
		p-value	OR	95% Confidence Interval for OR		p-value.	OR	95% Confidence Interval for OR	
				Lower Bound	Upper Bound			Lower Bound	Upper Bound
Independent variables during hospitali-	sex: F	0.269	0.779	0.500	1.214	0.324	0.692	0.334	1.437
	Waist circumference	0.411	0.979	0.931	1.030	0.428	0.970	0.901	1.045
	LDL-C	0.897	0.987	0.807	1.207	0.845	0.968	0.699	1.341
	HDL-C	0.012	0.539	0.332	0.875	0.000	0.134	0.043	0.415
	Triglycerides	0.915	0.985	0.750	1.295	0.027	1.419	1.041	1.936
	Total cholesterol	0.808	0.978	0.816	1.171	0.758	1.046	0.785	1.393
	BMI	0.002	1.099	1.036	1.165	0.002	1.142	1.051	1.242

Table S4. Multivariate Multinomial Logistic Regression: reference category Normoglycemic (NG).

	Prediabetes (IFG or IGT)				DM after hospitalization			
	p-value.	OR	95% Confidence Interval for OR		p-value	OR	95% Confidence Interval for OR	
			Lower Bound	Upper Bound			Lower Bound	Upper Bound
HDL-C	0.156	0.647	0.355	1.180	0.004	0.145	0.038	0.546
BMI	0.004	1.114	1.035	1.198	0.065	1.102	0.994	1.223

Figure S1. The distribution of diagnostic categories according to gender.

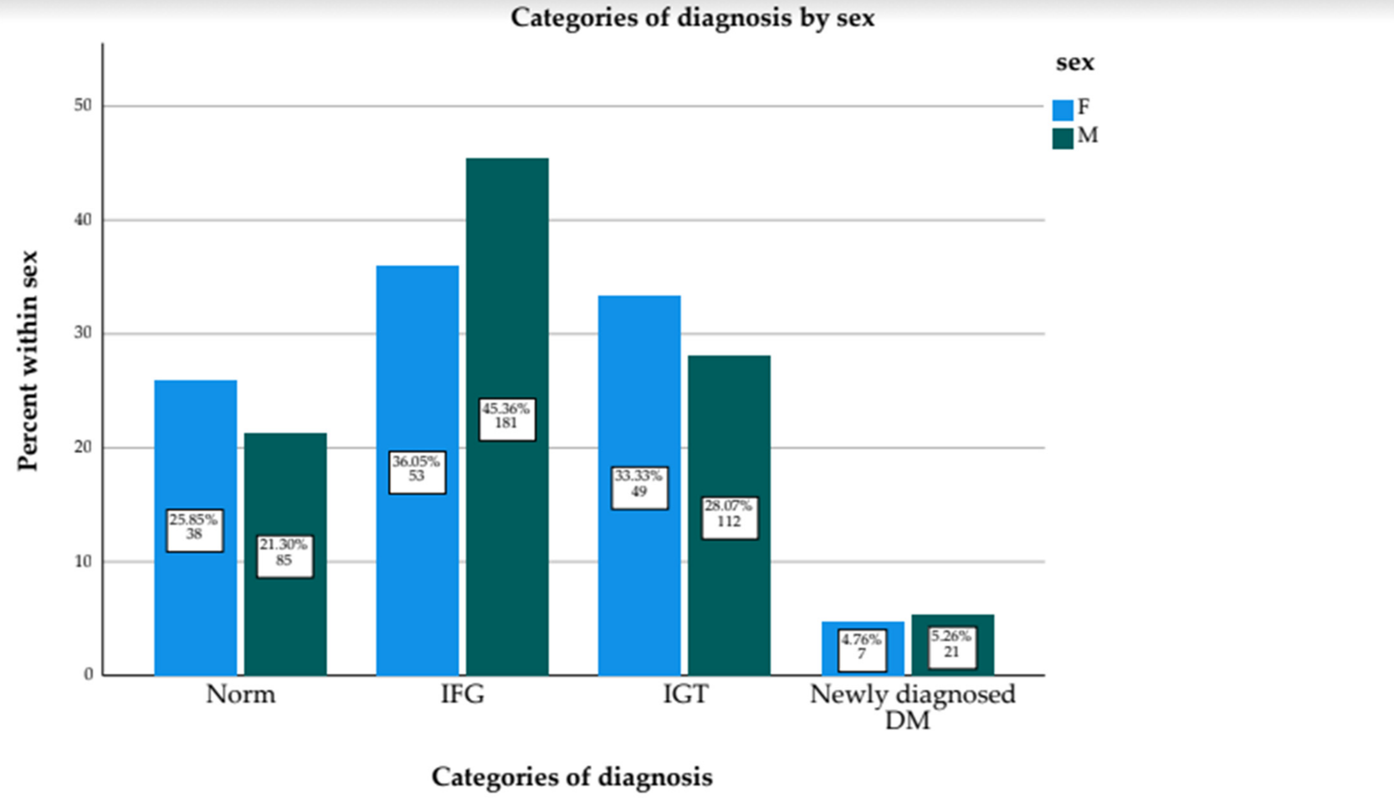

Categories of diagnosis by sex
sex
F
M
Percent within sex
0
10
20
30
40
50
25.85% 38
21.30% 85
36.05% 53
45.36% 181
33.33% 49
28.07% 112
4.76% 7
5.26% 21
Norm
IFG
IGT
Newly diagnosed DM
Categories of diagnosis
